# LncRNA MALAT1 accelerates non‐small cell lung cancer progression via regulating miR‐185‐5p/MDM4 axis

**DOI:** 10.1002/cam4.3570

**Published:** 2020-11-04

**Authors:** Dan Wang, Suhong Zhang, Min Zhao, Fengling Chen

**Affiliations:** ^1^ Department of Respiratory and Critical Medicine The Huaihe Hospital of Henan University Kaifeng Henan China; ^2^ Department of Urology The Huaihe Hospital of Henan University Kaifeng Henan China

**Keywords:** MALAT1, MDM4, miR‐185‐5p, NSCLC

## Abstract

Non‐small cell lung cancer (NSCLC) is the commonest malignancy with high death rate around the world. LncRNA metastasis‐associated lung adenocarcinoma transcript 1 (MALAT1) is greatly overexpressed in multifarious cancers, including NSCLC. However, the precise mechanism of MALAT1 in NSCLC tumorigenesis is blurry. This paper aims to investigate the theory of MALAT1 action in NSCLC progression. The levels of MALAT1, microRNA (miR)‐185‐5p, and mouse double minute 4 protein (MDM4) were measured by quantitative real‐time polymerase chain reaction (qRT‐PCR) or western blot. Cell proliferation and apoptosis were, respectively, determined by 3‐(4, 5‐dimethyl‐2‐thiazolyl)‐2, 5‐diphenyl‐2‐H‐tetrazolium bromide (MTT) assay, and flow cytometry. Cell migratory and invasive abilities were inspected by transwell assay. The binding relationship between miR‐185‐5p and MALAT1 or MDM4 was confirmed by dual‐luciferase reporter assay. The impacts of MALAT1 on tumor growth in vivo were measured by a xenograft experiment. We found MALAT1 and MDM4 were upregulated and MALAT1 positively regulated the MDM4 expression. MALAT1 and MDM4 deletion significantly hindered the proliferation, metastasis, and expedited the apoptosis of NSCLC cells. MDM4 overexpression partly overturned the influence of MALAT1 downregulation on cell development. Moreover, miR‐185‐5p, as a target of MALAT1, could directly target MDM4, and miR‐185‐5p upregulation exerted inhibitory effects on NSCLC cells. Besides, knockdown of MALAT1 inhibited tumor growth in vivo through miR‐185‐5p/MDM4 axis in NSCLC. Collectively, MALAT1 contributed to proliferation, migration, invasion, and impeded apoptosis by regulating the MDM4 expression mediated by miR‐185‐5p in NSCLC cells.

## Graphical abstract

This research showed high expression of MALAT1 in NSCLC tissues and cells. Moreover, knockdown of MALAT1 suppressed NSCLC progression via inhibiting cell proliferation, migration and invasion while facilitating apoptosis, possibly by up‐regulating miR‐185‐5p and decreasing MDM4 expression in NSCLC. Therefore, this study might elucidate a new regulatory mechanism for understanding malignant NSCLC and provide novel potential therapeutic targets for NSCLC treatment.

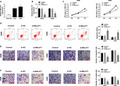

## 1 INTRODUCTION

Globally, as a member of the most familiar virulent tumors, lung cancer has the high occurrence rate and death rate,^1^ among which approximately 85% of cases are non‐small cell lung cancer (NSCLC).^2^, ^3^ NSCLC is often diagnosed in its late stages in which therapeutic options such as operative treatment are limited.^4^ Although noticeable improvements have been acquired in diagnosing and treating NSCLC, the 5‐year survival rate of patients is still very low.^5^ Hence, it is urgently necessary to seek for novel therapeutic targets and further explore the underlying action theory of NSCLC.

Long noncoding RNAs (lncRNAs) are a cluster of endogenous RNAs containing more than 200 nucleotides without protein‐coding potential.^6^ LncRNAs had aberrant expression and play vital parts in the onset and development of multifold cancers,^7^ including NSCLC.^8^ For example, lncRNACASC15 was high expressed and accelerated cell proliferation and metastasis by sponging miR‐130b‐3p in NSCLC.^9^ LncRNA PTAR contributed to NSCLC progression by targeting and inactivating miR‐101.^10^ Besides, lncRNA metastasis‐associated lung adenocarcinoma transcript 1 (MALAT1) has been confirmed to be an oncogene and involved in the tumorigenesis of several tumors including NSCLC.^11^ Nevertheless, the function and exact mechanism of MALAT1 have not been fully elucidated in NSCLC.

In the last decade, microRNAs (miRNAs) have been reported to participate in cleavage or translational repression by targeting mRNAs in animals and plants.^12^ Furthermore, the abnormal expression of miRNAs is associated with lung cancer carcinogenesis and behavior.^13^ For instance, miR‐196b repressed lung cancer cell propagation and movability via Runx2.^14^ MiR‐30e impeded cell proliferative and invasive capacities in NSCLC via directly sponging SOX9.^15^ In addition, increasing studies suggested that miR‐185‐5p served as an antineoplastic factor in hepatocellular carcinoma,^16^ glioma,^17^ and oral squamous cell carcinoma.^18^ A study has suggested that miR‐185‐5p was related to cisplatin resistance and cell growth of NSCLC cells.^19^ Whereas, the interaction between MALAT1 and miR‐185‐5p in NSCLC remains enigmatic.

Mouse double minute 4 (MDM4) is an essential opposite regulator of the P53 tumor suppressor, which has been reported to be upregulated and drive the malignancy of some cancers.^20^ For example, previous research revealed that MDM4 was significantly overexpressed in renal cancer and impeded the tumor progression.^21^ However, the role of MDM4 and the specific molecular mechanism in NSCLC needs to be further explored.

Herein, we measured the abundances of MALAT1 and MDM4 in NSCLC tissues and cells. Moreover, we probed into the function of MALAT1 and MDM4 in the development of NSCLC and confirmed the linear regulatory network of MALAT1/miR‐185‐5p/MDM4 axis.

## 2 MATERIALS AND METHODS

### 2.1 Clinical tissue samples

Thirty paired tumor tissues and adjacent normal tissues were provided by Huaihe Hospital of Henan University. All patients involved in this study, who had given informed consents did not receive any treatment before surgical resections. Meanwhile, the present research acquired a permission from the Ethics Committee of Huaihe Hospital of Henan University.

### 2.2 Cell culture

The normal lung bronchial epithelial cell line (BEAS‐2B) and lung cancer cell lines (A549 and H460) were purchased from American Type Culture Collection (ATCC). BEAS‐2B and A549 cells were maintained in Dulbecco's Modified Eagle's Medium (DMEM; Gibco), while H460 cells were cultivated in RPMI‐1640 medium (Gibco). Above media were all supplemented with 10% fetal bovine serum (FBS; Gibco). All these cells were trained in a 37°C humidified incubator under 5% CO_2_.

### 2.3 Quantitative real‐time polymerase chain reaction (qRT‐PCR)

For the separation of total RNA, TRIzol (Invitrogen) was utilized. Then, Nanodrop 2000 (Thermo Scientific) was utilized to quantity the isolated RNA. Next, the complementary DNA (cDNA) for MALAT1 and MDM4 was transcribed from RNA via Prime Script RT reagent Kit (Takara), while for miR‐185‐5p, the cDNA was transcribed with One Step Prime Script miRNA cDNA Synthesis Kit (Takara). Afterward, qRT‐PCR was performed for the amplification reaction using the SYBR Green Real‐Time PCR Master Mix (Thermo Scientific). Relative levels of target molecules mRNA were quantified in triplicate and calculated using the equation 2^−ΔΔCt^. GAPDH or U6 RNA was served as an internal parameter. The primers were displayed as follows:

MALAT‐1 forward (F), 5′‐GGTAACGATGGTGTCGAGGTC‐3′ and reverse (R), 5′‐CCAGCATTACAGTTCTTGAACATG‐3′;

miR‐185‐5p F, 5′‐GAAGGATCCGCATGAGAGGGTGTTGGAATGC‐3′ and R, 5′‐GGAGAATTCGTGCAGGGGCAGCAGACC‐3′;

MDM4 F, 5′‐CTCAGTGTCAACATCTGACAG‐3′ and R, 5′‐CATATGCTGCTCCTGCTGATC‐3′;

U6 F, 5′‐AACGCTTCACGAATTTGCGT‐3′ and R, 5′‐CCAAGCTTATGACAGCCATCATC‐3′;

GAPDH F, 5′‐TGAGATCAACGTGTTCCAGTG‐3′ and R, 5′‐ACCAGATGAAATGTGCCCC‐3′.

### 2.4 Western blot assay

Total protein was obtained with the cell lysis buffer (Abcam) and quantified using Bradford method. The protein was isolated by 10% sodium dodecyl sulfate‐polyacrylamide gel electrophoresis (SDS–PAGE) and then, transferred onto polyvinylidene fluoride (PVDF) membrane (Millipore). After sealed with 5% skim milk at room temperature for 1 h, the membrane was added with primary antibodies against MDM4 (1:2000; Abcam) (ab222905) and GAPDH (1:1000; Abcam) (ab9483) for 24 h at 4°C. Then, the membrane was added with secondary antibody labeled with horseradish peroxidase (HRP) (1:5000; Abcam) (ab205718). Finally, enhanced chemiluminescent (ECL) detection reagents (Beyotime Biotechnology) was utilized to make the protein bands visualization, and the signal intensity of the interest protein was analyzed by ImageJ software (NIH).

### 2.5 Cell transfection

Small interfering RNA‐(siRNA) targeting MALAT1 (si‐MALAT1), siRNA‐targeting MDM4 (si‐MDM4) and corresponding negative control (si‐NC), lentiviral particles harboring shRNA targeting XIST (sh‐XIST), and nontarget oligonucleotide sequence (sh‐NC) were structured by Genepharma. The MALAT1 and MDM4 overexpression vectors (MALAT1 and MDM4) and negative control pGL3 empty vector (pcDNA), as well as miR‐185‐5p mimics (miR‐185‐5p) and mimics negative control (miR‐NC) were obtained from Biomics Biotechnologies. All above plasmids or oligonucleotides were transduced into NSCLC cells using Lipofectamine 3000 reagent (Invitrogen) according to the user guide.

### 2.6 Cell proliferation assay

Briefly, transfected cells were planted in 96‐well plates (Sangon Biotech) and cultivated for indicated time points, severally. Then, 20 μl of 3‐(4, 5‐dimethyl‐2‐thiazolyl)‐2, 5‐diphenyl‐2‐H‐tetrazolium bromide (MTT, Sangon Biotech) was appended to each well and the admixture, followed by incubation at 37°C for 4 h. Whereafter, 150 μl dimethyl sulfoxide (DMSO; Sangon Biotech) was administrated to dissolve the formazan product. The value of optical density (OD) at 490 nm was determined by Multiskan Ascent Microplate Reader (Thermo Scientific).

### 2.7 Apoptosis assay

Transfected cells were suspended and stained with 5 μl Annexin V‐fluorescein isothiocyanate (FITC, BD Biosciences) and 5 μl propidium iodide (PI, BD Biosciences) for 25 min at 37°C after 48 h post‐transfection. At last, a FACScan flow cytometer with CellQuest3.0 software (BD Biosciences) was employed to detect and analyze the apoptotic cells.

### 2.8 Transwell assays

For migration assay, the cells were incubated for 48 h post‐transfection and then, seeded on the membrane surface of the upper chamber (24‐well, Corning, Corning, NY, USA) coated without Matrigel (BD Biosciences), whereas the lower chamber (Corning) was added with DMEM with 10% FBS to undertake the nutritional attractant. For the invasion assay, the whole process was similar to migration assay except that the membrane of upper chamber was added with Matrigel. After 48 h, the cells which had migrated or invaded into the bottom surface of the membrane were dyed with crystal violet (0.5% w/v) for 30 min after the fixation with methanol. The invaded or migrated cells were counted using a Countess automatic cell counter (Invitrogen) in five randomly chosen fields under inverted microscope (Nikon TE‐300).

### 2.9 Dual‐luciferase reporter assay

The potential targeted sequence between miR‐185‐5p and MALAT1 or MDM4 was predicted by starBase v2.0 or TargetScan Human 7.2, correspondingly. The wild‐type luciferase reporter vectors (WT‐MALAT1 and WT‐MDM4) and corresponding mutant vectors (MUT‐MALAT1 and MUT‐MDM4) containing the putative binding sequence of miR‐185‐5p were constructed based on psiCHECK2 vector (Promega). A549 and H460 cells were cultivated in 96‐well plates and introduced with constructed vectors and miR‐185‐5p or miR‐NC. After transfection for 48 h, the luciferase intensities of the cell lysates from harvested cells were detected with Dual‐Luciferase Reporter Assay System (Promega).

### 2.10 Xenograft mouse model

BALB/c nude mice (5 weeks old, *n* = 5 per group) acquired from Shanghai Experimental Animal Center (Shanghai, China) were used to construct xenograft mouse models. A549 cells infected with lentivirus harboring sh‐MALAT1 or negative control sh‐NC were subcutaneously inoculated into the left flank of nude mice. The maximum length (recorded as “*a*”) and minimum length (recorded as “*b*”) of the tumor was measured to monitor tumor growth every 4 days after 7 days of first injection. Then, tumor volume (recorded as “*V*”) was calculated through the formula: *V* = *ab* ^2^/2. The resected xenografts were weighed after all mice were killed at 28 days. The animal research was ratified by the Animal Care and Use Committee of XXXX.

### 2.11 Statistical analysis

The statistical analysis of all data was conducted by SPSS 21.0 software and appeared as mean ± standard deviation. The data were replicated for at least three parallel experiments. For the difference comparison, Student's *t*‐test and one‐way analysis of variance were adopted. Spearman correlation analysis was used for examination of the correlation. *p* < 0.05 was deemed to be statistically significant.

## 3 RESULTS

### 3.1 MALAT1 and MDM4 were highly expressed in NSCLC tissues

**FIGURE 1:**
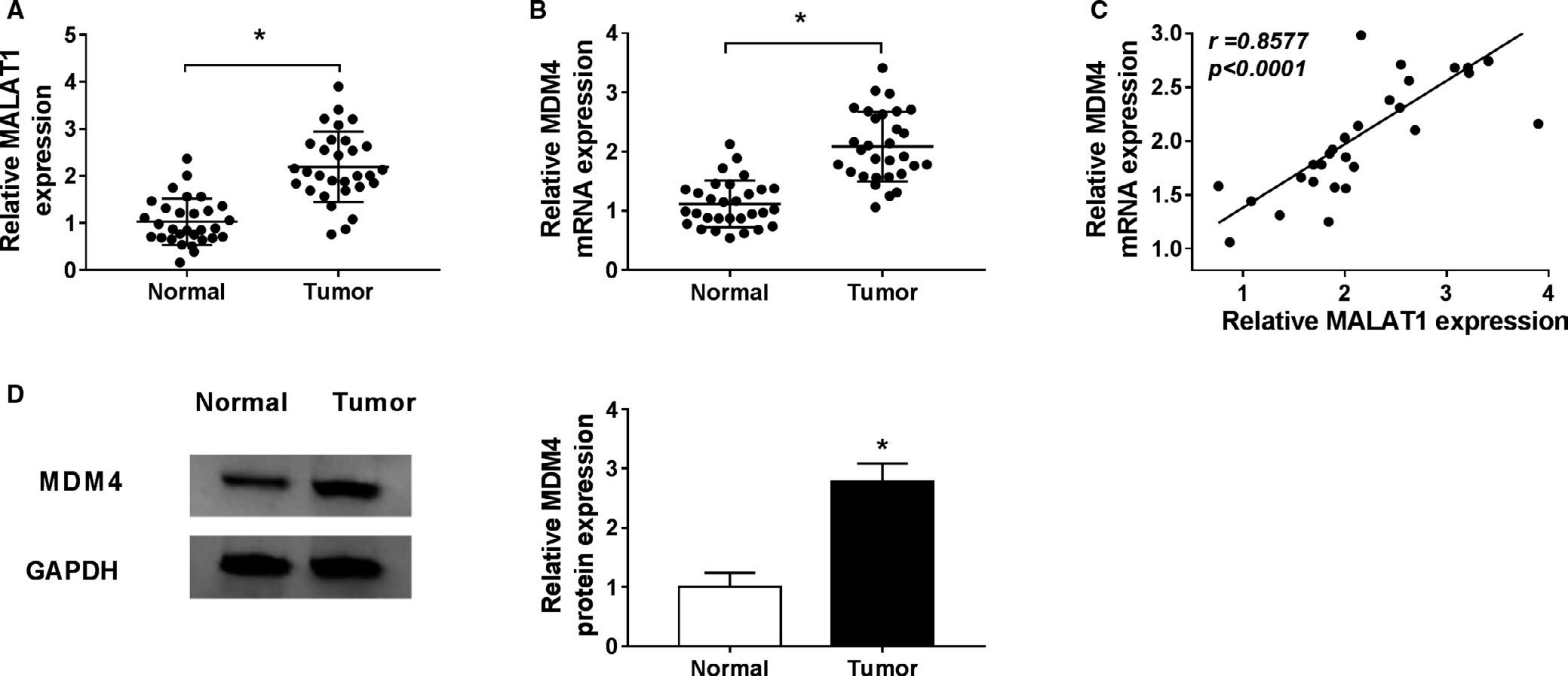
The expression of MALAT1 and MDM4 was raised in NSCLC tissues. (A and B) The relative expression of MALAT1 and MDM4 mRNA was detected by qRT‐PCR in 30 paired NSCLC tumor tissues and corresponding normal tissues. (C) The interaction between MALAT1 and MDM4 in tumor tissues was attested by spearman's correlation analysis. (D) The enrichment of MDM4 protein was also certified in tumor tissues and adjacent normal tissues by western blot.*p < 0.05

First, qRT‐PCR or western blot was proceeded to measure the relative expression of MALAT1 and MDM4. As depicted in Figure 1A, compared to normal tissues, the level of MALAT1 was distinctly increased in 30 paired NSCLC tumor tissues, and the same result was observed in the mRNA and protein abundances of MDM4 (Figure 1B and D). Furthermore, Spearman's correlation analysis suggested that MALAT1 expression was positively correlated with the MDM4 level (Figure 1C). The data implied that MALAT1 and MDM4 might play vital parts in NSCLC.

### 3.2 MALAT1 silence inhibited the proliferation, migration, invasion, and promoted the apoptosis in NSCLC cells

**FIGURE 2:**
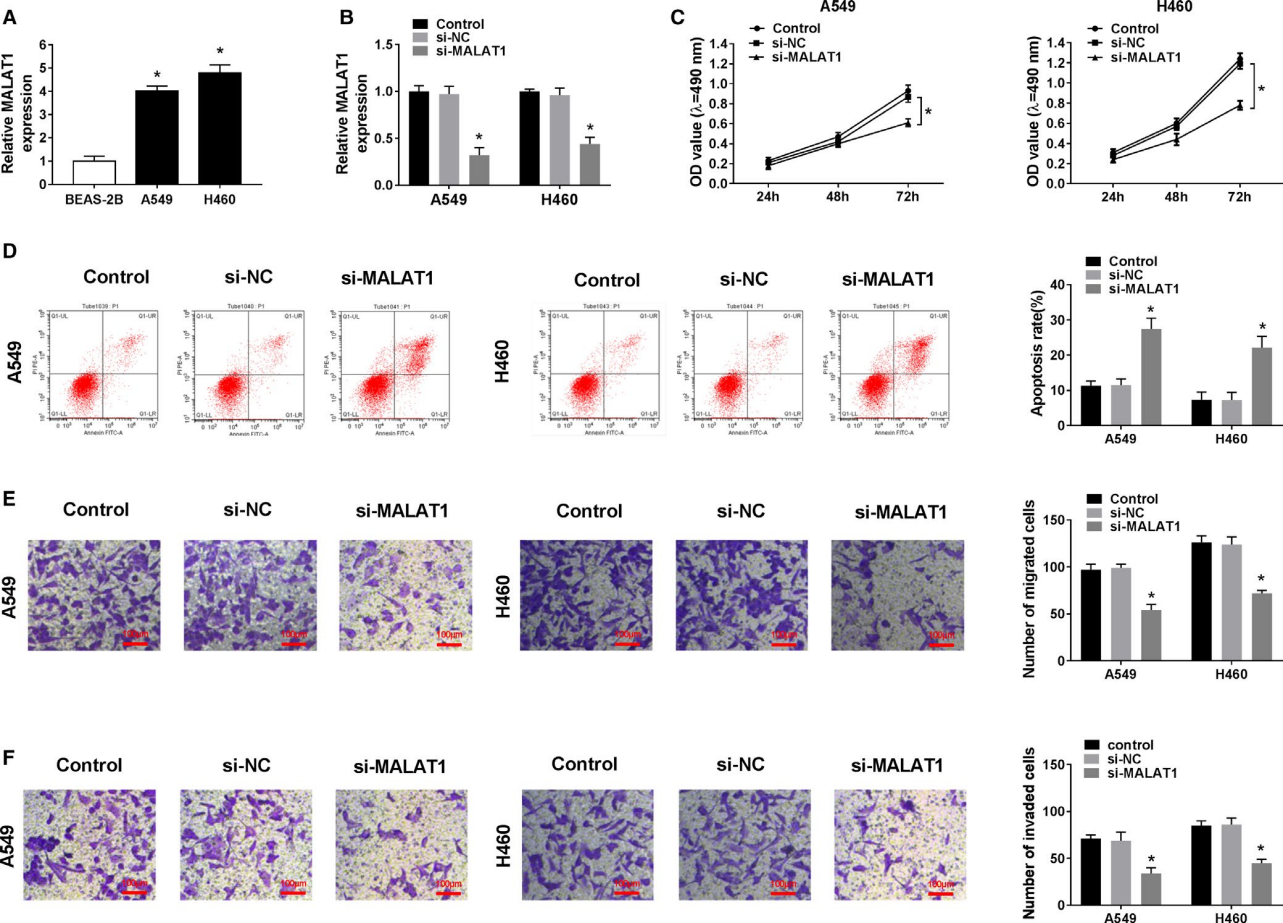
Silence of MALAT1 inhibited the progression of NSCLC cells. (A) The relative expression of MALAT1 in NSCLC cell lines (A549 and H460) and normal cell line BEAS‐2B was assessed by qRT‐PCR. (B–F) A549 and H460 cells were introduced with si‐MALAT1 or negative control si‐NC. (B) The efficiency of MALAT1 knockdown was tested by qRT‐PCR. (C) The proliferation was investigated by MTT assay. (D) The apoptosis was examined by flow cytometry. (E–F) The migration and invasion were evaluated by transwell assay. *p < 0.05

In accordance with the result of MALAT1 expression in NSCLC tumor tissues, the enrichment of MALAT1 was also increased in NSCLC cells (A549 and H460) relative to normal BEAS‐2B cells (Figure 2A). To explore the role of MALAT1, A549 and H460 cells were transduced with si‐MALAT1 or control si‐NC. MALAT1 level was reduced in si‐MALAT1‐transfeced NSCLC cells versus that of si‐NC and Control group, implying the successful transfection (Figure 2B). MTT assay manifested that cell proliferation was notably inhibited in A549 and H460 cells after MALAT1 silence, while the apoptosis rate was visibly raised, which was verified by flow cytometry (Figure 2C and D). Compared with si‐NC and Control group, transwell assay revealed that cell migrated and invaded abilities were strongly declined caused by the deficiency of MALAT1 (Figure 2E and F). These explorations established that knockdown of MALAT1 impeded the malignant behaviors of NSCLC cells.

### 3.3 Knockdown of MDM4 suppressed cell growth in NSCLC cells

**FIGURE 3:**
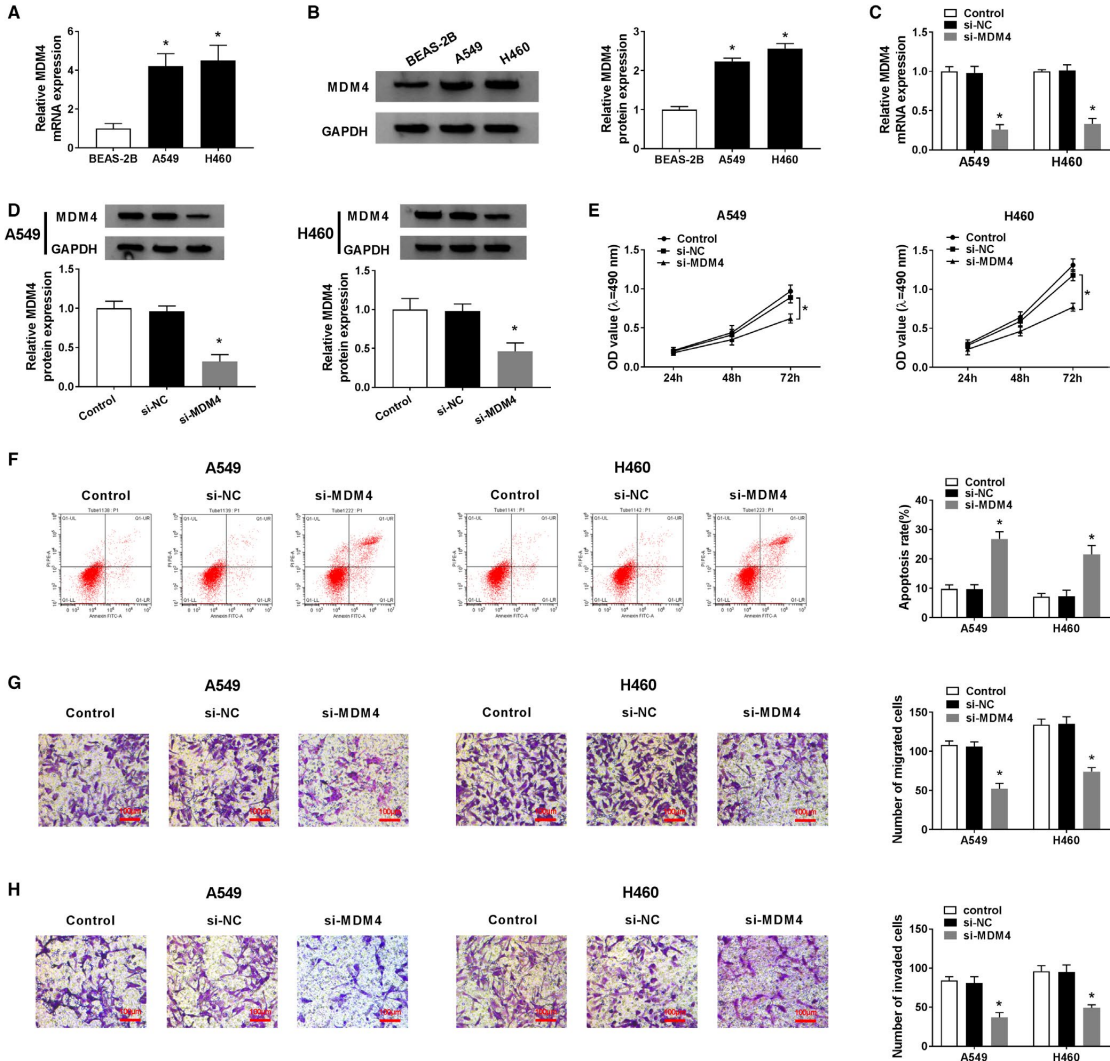
Knockdown of MDM4 impeded cell malignant behaviors in NSCLC cells. (A and B) The relative MDM4 mRNA and protein expressions in BEAS‐2B, A549, and H460 cells were checked by qRT‐PCR or western blot. (C–G) A549 and H460 cells were transduced with si‐MDM4 or si‐NC. (C and D) The knockdown efficiency of MDM4 was verified by qRT‐PCR and western blot. (E) The proliferation was explored by MTT assay. (F) The apoptosis was appraised by flow cytometry. (G and H) The migratory and invasive capacities were tested by transwell assay. *p < 0.05

In order to identify the MDM4 expression in NSCLC cells, qRT‐PCR and western blot assays were conducted. The result suggested that MDM4 level was distinctly elevated in A549 and H460 cells compared to that of BEAS‐2B cells (Figure 3A and B). Then A549 and H460 cells were introduced with si‐MDM4 or si‐NC to probe into the function of MDM4 in NSCLC cells. The knockdown efficiency was certified by examining the MDM4 level in NSCLC cells with si‐MDM4 or si‐NC transfection, in which MDM4 was downregulated after MDM4 silence (Figure 3C and D). Meanwhile, cell proliferation, migration, and invasion were all overtly restricted due to the knockdown of MDM4 (Figure 3E, G and H); in contrast, cell apoptosis was notably enhanced after knockdown of MDM4 (Figure 3F). Together, these data pointed out that MDM4 acted as a cancer‐promoting gene in NSCLC cells.

### 3.4 MALAT1 regulated the expression of MDM4 in NSCLC cells

**FIGURE 4:**
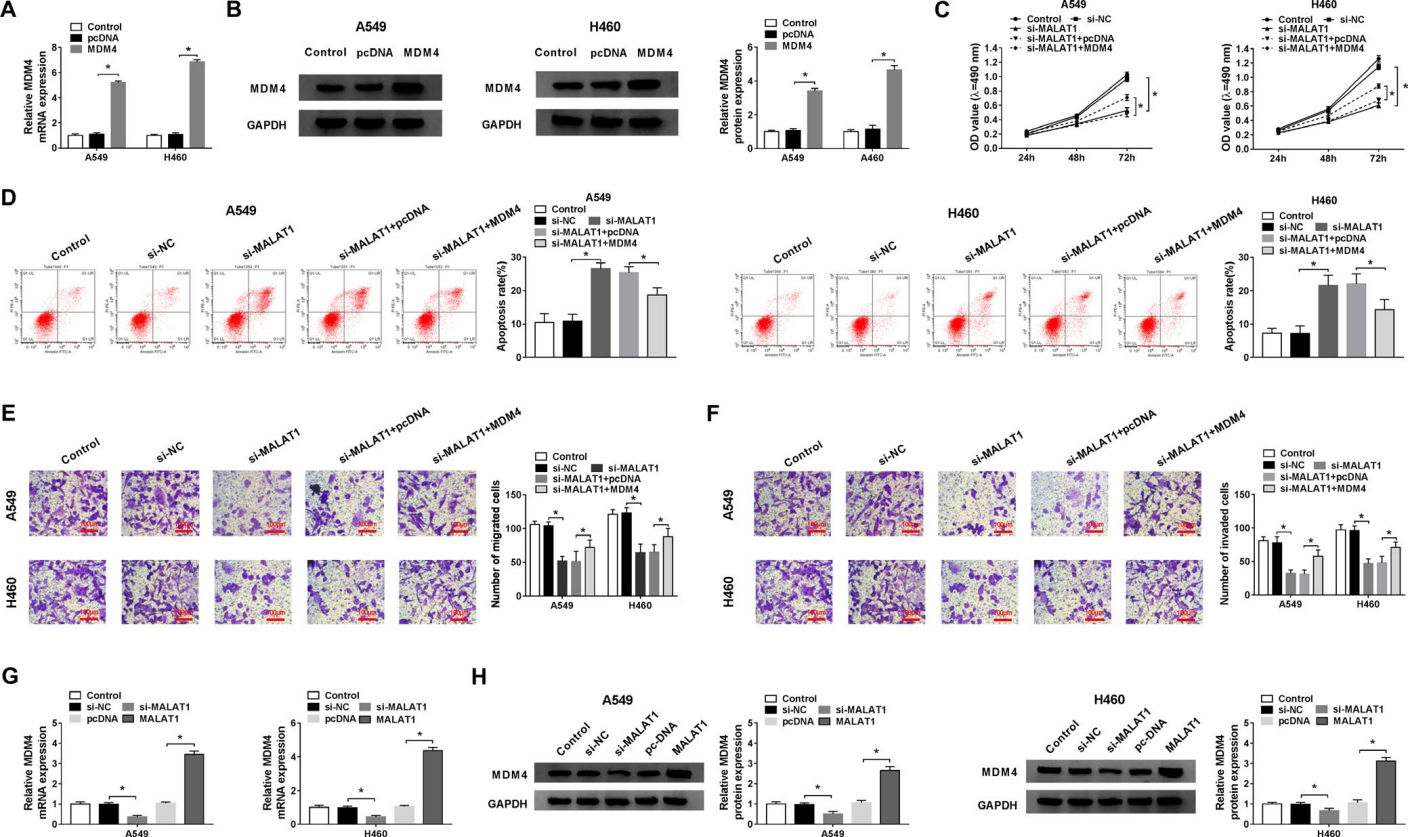
MALAT1 modulated MDM4 expression in NSCLC cells. (A and B) The overexpression efficiency of MDM4 was demonstrated. (C–F) A549 and H460 cells were introduced with si‐NC, si‐MALAT1, si‐MALAT1+pcDNA, or si‐MALAT1+ MDM4. (C) The proliferation was tested by MTT assay. (D) The apoptosis was checked by flow cytometry. (E and F) The migratory and invasive abilities were examined by transwell assay. (G and H) The expression of MDM4 was explored after MALAT1 knockdown or overexpression in A549 and H460 cells via qRT‐PCR and western blot, correspondingly. *p < 0.05

On the basis of the aforementioned findings, the relation between MALAT1 and MDM4 was further studied through transfection with si‐MALAT1, si‐MALAT1+ MDM4, and matched negative controls into A549 and H460 cells. The mRNA and protein abundances of MDM4 in A549 and H460 cells after MDM4 overexpression were strongly augmented relative to pcDNA and Control group, hinting successful transfection (Figure 4A and B). The inhibitory effects of MALAT1 downregulation on cell proliferative and transferred abilities and the accelerating effects on cell apoptotic capacity were all partly reversed by MDM4 overexpression (Figure 4C–F). Moreover, MDM4 levels were evidently decreased after MALAT1 knockdown, while they were extremely increased after overexpression of MALAT1 in A549 and H460 cells (Figure 4G and H). From these data, it could be concluded that MALAT1 directly regulated MDM4 and there was a positive relationship between them in NSCLC cells.

### 3.5 MiR‐185‐5p impeded the progression of NSCLC cells

**FIGURE 5:**
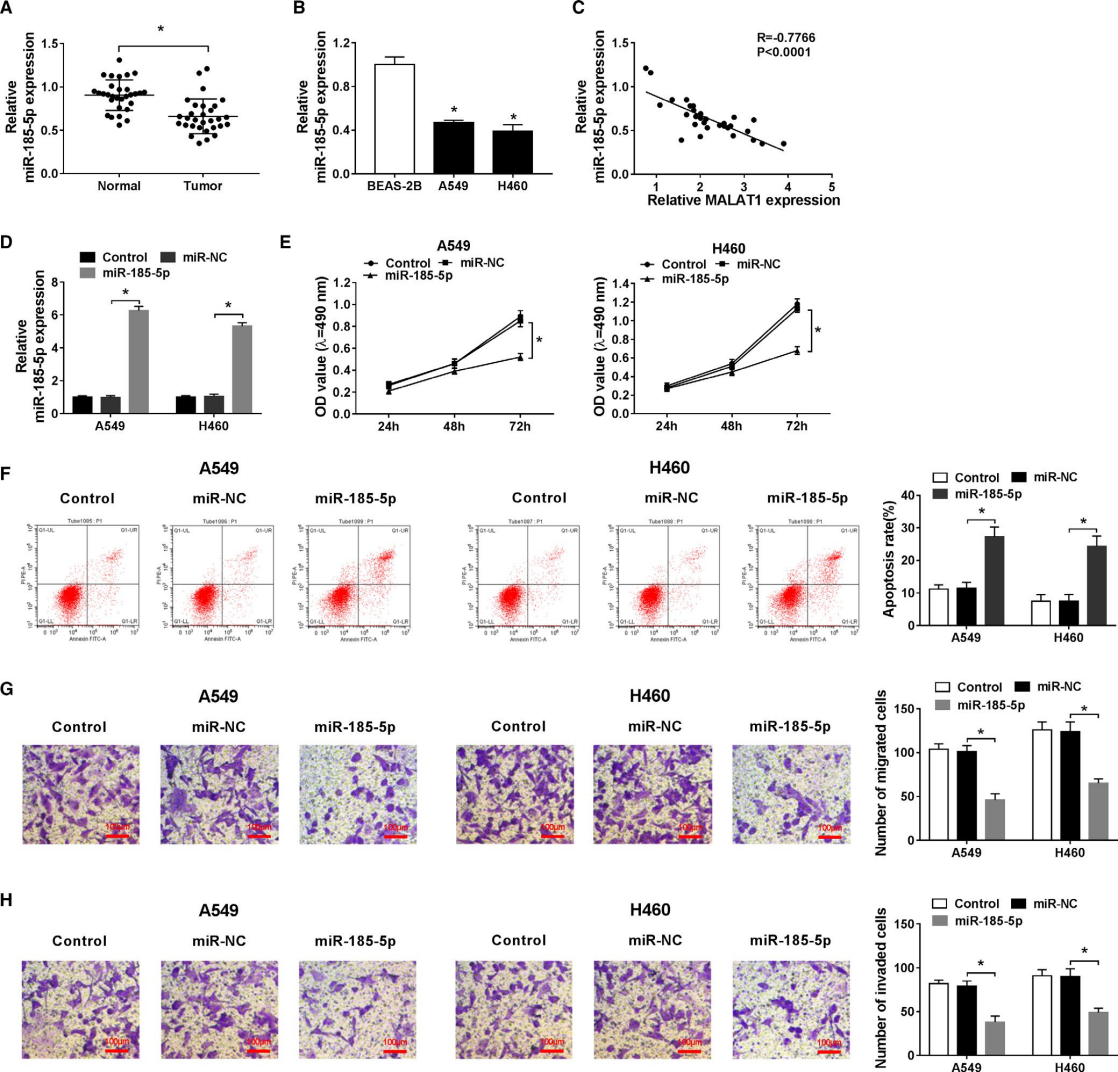
MiR‐185‐5p hindered the progression of NSCLC cells. (A) The expression of miR‐185‐5p in 30 paired NSCLC tumor tissues and corresponding normal tissues was measured by qRT‐PCR. (B) The expression of miR‐185‐5p in BEAS‐2B, A549, and H460 cells was tested by qRT‐PCR. (C) The relationship between MALAT1 and miR‐185‐5p in tumor tissues was demonstrated by spearman's correlation analysis. (D) The overexpression efficiency of miR‐185‐5p in A549 and H460 cells was verified. (E–H) The proliferation (E), apoptosis (F), movability (G and H) of A549 and H460 cells transduced with miR‐185‐5p or miR‐NC were detected by MTT assay, flow cytometry, and transwell assay, respectively.*p < 0.05

In addition to the above findings, the level of miR‐185‐5p was discovered to be downregulated in the NSCLC tumor tissues and cells (Figure 5A and B). Meanwhile, the enrichment of miR‐185‐5p was inversely related to MALAT1 level (Figure 5C). To affirm the role of miR‐185‐5p in NSCLC, gain‐of‐function experiment was carried out. The result certified that miR‐185‐5p was immensely raised which demonstrated the successful transfection (Figure 5D). Interestingly, the role of miR‐185‐5p was consistent with that of si‐MALAT1 in NSCLC cells. More specifically, overexpression of miR‐185‐5p overtly impeded cell proliferation and metastasis relative to control and miR‐NC group (Figure 5E, G and H), whereas expedited the cell apoptotic rate in A549 and H460 cells (Figure 5F). Overall, the above results revealed that miR‐185‐5p might be related to MALAT1 in the growth of NSCLC cells.

### 3.6 MALAT1 regulated MDM4 expression by targeting miR‐185‐5p

**FIGURE 6:**
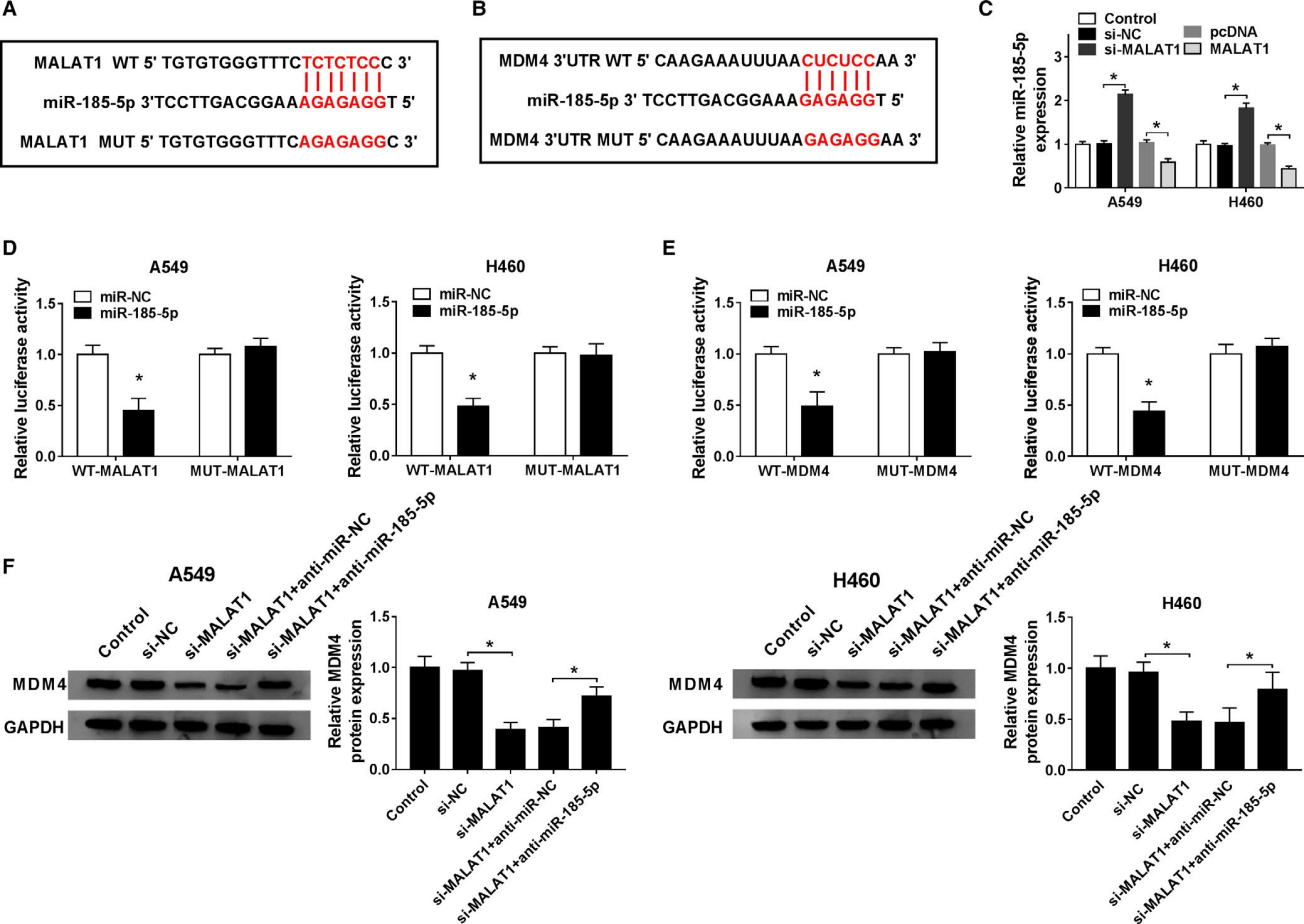
MALAT1 regulated MDM4 level by targeting miR‐185‐5p. (A) The binding sites between MALAT1 and miR‐185‐5p were predicted by starBase v2.0. (B) The complementary sequence between miR‐185‐5p and MDM4 was forecasted by TargetScan Human 7.2. (C) The enrichment of miR‐185‐5p in A549 and H460 cells transduced with si‐MALAT1, MALAT1, or corresponding negative control was assessed. (D and E) The relationship between miR‐185‐5p and MALAT1 or MDM4 was corroborated by dual‐luciferase reporter assay. (F) The expression of MDM4 at the protein level in A549 and H460 cells introduced with si‐MALAT1, anti‐miR‐185‐5p + si‐MALAT1, or corresponding negative control was measured by western blot. *p < 0.05

On basis of above experimental findings referring to the characters of MALAT1, miR‐185‐5p and MDM4 in NSCLC cells, further studies were carried out as follows. Above all, bioinformatics tools were adopted to forecast whether there were complementary sites among MALAT1, miR‐185‐5p and MDM4. Fortunately, MALAT1 was predicted to have binding sites with miR‐185‐5p using starBase v2.0 online database (Figure 6A), and there was binding sequence between miR‐185‐5p and MDM4 as well by TargetScan Human 7.2 (Figure 6B). Then, the combination about miR‐185‐5p and MALAT1 or MDM4 was further attested via dual‐luciferase reporter assay. Conformably, the luciferase intensity was overtly declined caused by the introduction of miR‐185‐5p and WT‐MALAT1 or WT‐MDM4, while it had no difference in cells transduced with MUT‐MALAT1 or MUT‐MDM4 and miR‐185‐5p or miR‐NC (Figure 6D and E). Meanwhile, miR‐185‐5p was upregulated due to the transfection of si‐MALAT1, whereas it was downregulated by MALAT1 overexpression (Figure 6C). Furthermore, Functional experiment attested that the protein expression of MDM4 was declined in A549 and H460 cells introduced with si‐MALAT1, while the decrease was again recovered by cotransfection with anti‐miR‐185‐5p and si‐MALAT1 (Figure 6F). Collectively, these findings revealed that MALAT1 might modulate NSCLC development by regulating the expression of MDM4 mediated by miR‐185‐5p.

### 3.7 Knockdown of MALAT1 inhibited tumor growth in vivo

**FIGURE 7:**
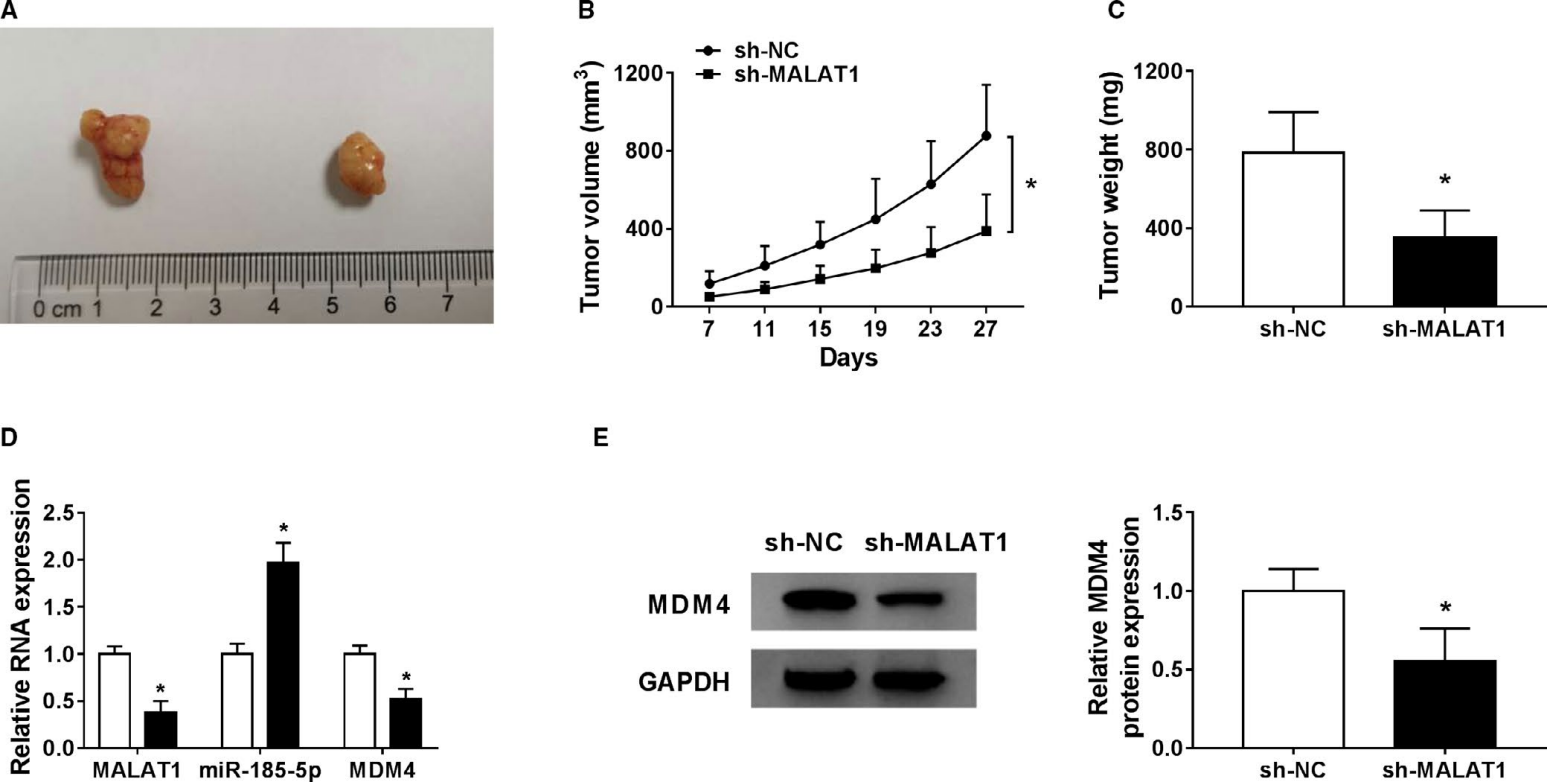
MALAT1 silence impeded tumor growth in vivo. A549 cells (2 × 10^6^/0.2 ml PBS) stably infected with sh‐MALAT1 or sh‐NC were subcutaneously inoculated into the nude mice. (A) The tumor images were taken. (B) After 7 days implantation, tumor volume measurement was conducted every 4 days. (C) Xenograft tissues were weighted and the average weight was calculated. (D) The levels of MALAT1, miR‐185‐5p, and MDM4 in tumor tissues were examined by qRT‐PCR. (E) The protein expression of MDM4 in tumor tissues was inspected by western blot. *p < 0.05

To further investigate whether knockdown of MALAT1 could attenuate the progression of NSCLC in vivo, A549 cells stably transduced with sh‐MALAT1 or sh‐NC were subcutaneously inoculated into the nude mice. Twenty‐seven days later, all mice were sacrificed and the xenografts were resected (Figure 7A). Data suggested that the tumor volume and weight of xenografts in sh‐MALAT1 group were dramatically smaller than that in sh‐NC groups (Figure 7B and C). The levels of MALAT1 and MDM4 in sh‐MALAT1 group were declined while the level of miR‐185‐5p was increased (Figure 7D and E). These results revealed that MALAT1 inhibited tumor growth via affecting MDM4/miR‐185‐5p pathway in vivo.

## 4 DISCUSSION

NSCLC is the common cause of cancer‐related death with high death rate globally.^2^ The dysregulated lncRNAs might be associated with the diagnosis and therapy of lung cancer.^22^ Also, lncRNAs have been demonstrated as underlying prognostic biomarkers for NSCLC.^23^ Increasing reports have indicated that MALAT1 can play parts in different tumors through working as a carcinogenic factor or anticancer factor due to the variation of tumor microenvironment.^24^ Previous research suggested that MALAT1 had a distinct incremental level in NSCLC.^11^, ^25^ Nevertheless, the role of MALAT1 in NSCLC development has not been completely elaborated. In the current study, the suppressive effect of MALAT1 silence on NSCLC development in vitro and in vivo was exhibited. Moreover, we gained insight into the competing endogenous RNA (ceRNA) network of MALAT1/miR‐185‐5p/MDM4 in NSCLC.

LncRNAs could serve as miRNA decoys to modulate the level of target genes.^26^ Previous research suggested that MALAT1 could accelerate cell transferability via targeting miR‐206 ^25^ and contribute to cell proliferation and hinder apoptosis via miR‐124/STAT3 axis in NSCLC.^11^ In accordance with these studies, this study revealed the high expression of MALAT1 in NSCLC, indicating that MALAT1 overexpression might be closely connected with the malignancy of NSCLC. Meanwhile, the loss‐of‐function experiments confirmed the anti‐proliferation, anti‐metastasis, and pro‐apoptosis roles of MALAT1 knockdown in NSCLC, and xenograft mouse model further verified the tumor‐promoter role of MALAT1 in vivo. All these data suggested that MALAT1 acted as a cancer‐promoting factor in the carcinogenesis of NSCLC.

As a p53 inhibitor, the study has shown that MDM4 is frequently overexpressed and may act as a remedial target in multiple human cancers.^27^ MDM4 could promote NSCLC progression acting as a target of miRNAs (such as miR‐1205^28^ and miR‐34a‐5p^29^) in NSCLC. In line with previous research, MDM4 was also demonstrated to be upregulated in NSCLC tissues and cells in this present paper, which was the same as the results of MALAT1. Simultaneously, the abundance of MDM4 had a positive correlation with MALAT1 level in NSCLC tissues, and knockdown of MDM4 impeded proliferation, migration, invasion, and enhanced the apoptosis. Interestingly, MDM4 overexpression could partly overturn the effects on proliferation, migration, invasion, and apoptosis induced by MALAT1 silence. Moreover, MDM4 was downregulated by MALAT1 knockdown and was upregulated by MALAT1 overexpression. These findings revealed that MALAT1 might regulate NSCLC progression by increasing MDM4 expression.

Growing evidence proved that miRNAs played crucial effects on the occurrence and development of human malignant tumors, including NSCLC.^30^ MiR‐185 could serve as a tumor inhibitor and induce a G1 cell cycle arrest in NSCLC cells.^31^, ^32^ Here, miR‐185‐5p level was attested to be lessened in NSCLC, and miR‐185‐5p upregulation manifested a depressant impact on proliferation, migration, invasion, and a facilitating effect on apoptosis. Importantly, dual‐luciferase reporter assay testified that miR‐185‐5p was targeted by MALAT1. The functional experiments disclosed that miR‐185‐5p was reversely modulated by MALfaAT1 in NSCLC cells, implying MALAT1 might participate in NSCLC progression via miR‐185‐5p. The miRNAs could modulate the gene expression by either mRNA degradation or translational suppression of target genes.^12^ It is well documented that miR‐185 could hinder the malignant behaviors of NSCLC by targeting KLF7^33^ or AKT1^31^. Furthermore, miR‐185‐5p mimics could restrain cell proliferation by sponging ABCC1.^19^ In the current study, we attested that miR‐185‐5p‐targeted MDM4 and MALAT1 overexpression could partly reverse the effects of miR‐185‐5p mimics on MDM4 level. On the whole, our findings confirmed that MALAT1 modulated the progression of NSCLC partly through the miR‐185‐5p/MDM4 axis.

In conclusion, this research showed elevated abundance of MALAT1 in NSCLC tissues and cells. Moreover, knockdown of MALAT1 suppressed NSCLC progression via inhibiting growth and metastasis, and facilitating apoptosis, possibly through upregulating miR‐185‐5p and decreasing MDM4 expression in NSCLC. Therefore, this study might elucidate a new regulatory mechanism for understanding malignant NSCLC and provide novel latent remedial targets for NSCLC therapy.

## ETHICS APPROVAL AND CONSENT TO PARTICIPATE

The present study was approved by the ethical review committee of the Huaihe Hospital of Henan University.

## PATIENT CONSENT FOR PUBLICATION

Not applicable.

## CONFLICT OF INTEREST

The authors declare that they have no financial conflict of interest.

## AUTHORS’ CONTRIBUTIONS

Conceptualization and Methodology: Suhong Zhang and Min Zhao; Formal analysis and Data curation: Dan Wang, Min Zhao, and Fengling Chen; Validation and Investigation: Dan Wang and Suhong Zhang; Writing—original draft preparation and Writing—review and editing: Dan Wang, Suhong Zhang, and Min Zhao; Approval of final manuscript: all authors.

## Data Availability

The analyzed data sets generated during the present study are available from the corresponding author on reasonable request.
